# Identification of Prognostic Gene Signatures by Developing a scRNA-Seq-Based Integration Approach to Predict Recurrence and Chemotherapy Benefit in Stage II–III Colorectal Cancer

**DOI:** 10.3390/ijms232012460

**Published:** 2022-10-18

**Authors:** Zixuan Wang, Kaiyuan Xing, Bo Zhang, Yanru Zhang, Tengyue Chai, Jingkai Geng, Xuexue Qin, Xinxin Zhang, Chaohan Xu

**Affiliations:** College of Bioinformatics Science and Technology, Harbin Medical University, No. 194, Xue-Fu Road, Nangang Region, Harbin 150081, China

**Keywords:** scRNA-seq, bulk data, CRC, prognostic signature

## Abstract

Prospective identification of robust biomarkers related to prognosis and adjuvant chemotherapy has become a necessary and critical step to predict the benefits of adjuvant therapy for patients with stage II–III colorectal cancer (CRC) before clinical treatment. We proposed a single-cell-based prognostic biomarker recognition approach to identify and construct CRC up- and down-regulated prognostic signatures (CUPsig and CDPsig) by integrating scRNA-seq and bulk datasets. We found that most genes in CUPsig and CDPsig were known disease genes, and they had good prognostic abilities in CRC validation datasets. Multivariate analysis confirmed that they were two independent prognostic factors of disease-free survival (DFS). Significantly, CUPsig and CDPsig could effectively predict adjuvant chemotherapy benefits in drug-treated validation datasets. Additionally, they also performed well in patients with CMS4 subtype. Subsequent analysis of drug sensitivity showed that expressions of these two signatures were significantly associated with the sensitivities of CRC cell lines to multiple drugs. In summary, we proposed a novel prognostic biomarker identification approach, which could be used to identify novel prognostic markers for stage II–III CRC patients who will undergo adjuvant chemotherapy and facilitate their further personalized treatments.

## 1. Introduction

Colorectal cancer (CRC) is the third most common cancer worldwide, with the second highest cancer mortality rate [1]. The survival outcomes of patients with different stages of CRC vary greatly, and the five-year survival rate of patients with stage I CRC is 93%, while the corresponding survival rates in stage II, III, and IV CRC patients are 70%, 60%, and 8%, respectively [2,3]. Clinically, patients with stage II–III CRC usually receive adjuvant chemotherapy to reduce the recurrence rate or prolong the recurrence time and improve survival [4,5]. Therefore, how to identify and establish robust biomarkers related to prognosis and adjuvant chemotherapy has become very necessary. A large number of studies based on bulk expression (microarray or RNA-seq detection) data had been carried out and provided a broad data basis for seeking these high-risk biomarkers. For example, Liu et al. developed and validated a robust prognostic signature with six genes (ELMSAN1, KRT33B, NDRG1, PPP1R13L, PPP2R1B, and WDYHV1). They found that the prognostic signature could accurately predict recurrence risk in stage II–III CRC patients and help optimize post-operative monitoring and treatment strategies [6]. Ren et al. established a CRC prognostic signature, which was named ERG signature and composed of AXL, TCFL5, KLK6, PDGFD, SOD2, UBD, FUT4, ACTB, RPL10A, and HNRNPK. They found that the ERG signature was an independent prognostic factor for stage II–III CRC patients, and survival analysis results demonstrated that high-risk patients had shorter recurrence and overall survival time than low-risk patients [7]. What is more, Song et al. developed a 44 gene pair–based signature (44-GPS), and they confirmed that 44-GPS could successfully predict post-operative recurrence risk of stage II–III CRC patients in different microarray data and RNA-seq data [8]. Although some achievements have been made in the field of discriminating such biomarkers, the samples of traditional bulk data detected by bulk data were usually mixed with a large number of non-cancer samples, including tumor microenvironment or paracancerous tissue, which interfered with the identification of prognostic gene markers and study of their functional mechanisms in stage II–III CRC patients.

Fortunately, with the rapid development of sequencing technology, single-cell RNA-sequencing (scRNA-seq) technology had improved the above-mentioned limitations of bulk data, and thus provided more powerful technical support for the study of prognostic gene markers in CRC patients. For example, based on CRC scRNA-seq data, Zheng et al. established a cancer-associated fibroblast (CAF)-related prognostic signature, including HSPB1, S100A13, PPP1R14A, CSRP2, TIPM2, CEBPD, TIMP1, SPINK1, and CXCL1. Univariate and multivariate Cox regression analysis showed that the signature was an independent prognostic indicator for predicting overall survival in CRC patients [9]. Tang et al. revealed the pervasive genomic variation in CRC stromal cells through scRNA-seq technology and found that BGN, RCN3, TAGLN, MYL9, and TPM2 could be used as specific biomarkers for CAF patients with poor prognosis [10]. Since current single-cell technologies cannot directly link cell types to clinical phenotypes of cancer patients, integrating single-cell data and bulk data to discover more accurate biomarkers is very important and necessitated. Some algorithms integrated scRNA-seq and bulk datasets have been generated, such as Scissor (Single-cell Identification of Subpopulations with Bulk Sample Phenotype Correlation) and BayesPrism (Bayesian cell proportion reconstruction inferred using statistical marginalization). The Scissor algorithm enables to systematically quantify the similarity between single-cell data and bulk data across single-cell sequencing data, bulk expression data and corresponding clinical phenotype information, thereby identifying the cell subgroups that are most relevant to a given phenotype group in the scRNA-seq data [11]. BayesPrism was designed by the Bayesian model for integrative analysis of scRNA-seq and bulk datasets. It used scRNA-seq data as prior information to infer cell type composition and gene expression in each bulk RNA-seq sample and can identify common malignant gene programs by removing gene expression in nonmalignant cells [12].

The combination of single cell data and bulk data can provide new insights into the identification of prognosis-related biomarkers, resulting in generating more reliable biomarkers that can be further used for the prediction of the benefits of adjuvant chemotherapy. Therefore, we proposed a single-cell-based prognostic marker recognition method by integrating two single-cell datasets (GSE132465, GSE144735) and three bulk datasets (GSE17538, GSE39582, and GSE37892). We used the Scissor algorithm embedded in our approach to correlate cancer cells in scRNA-seq data with prognosis phenotypes of stage II–III CRC patients in bulk validation data to construct two CRC prognostic signatures, common up-regulated signature (CUPsig) and common down-regulated signature (CDPsig). We then evaluated these two signatures in multiple perspectives, such as functional enrichment analysis, prognostic validation in multi-dataset, adjuvant chemotherapy analysis, CMS4 subtype analysis, and drug sensitivity analysis. Most of the genes in CUPsig and CDPsig were found to be CRC disease genes, and prognostic analysis results showed that CUPsig and CDPsig could effectively predict disease-free survival (DFS) in patients with stage II–III CRC. Multivariate analysis indicated that they were two independent prognostic factors. Notably, CUPsig and CDPsig still had good predictive effects for patients who have received adjuvant chemotherapy. In addition, our prognostic signatures also performed well in patients with CMS4 subtype. Additionally, through drug sensitivity analysis, we found that the expressions of CUPsig and CDPsig were closely related to the sensitivities of CRC cell lines to multiple drugs.

## 2. Results

### 2.1. Construction of a scRNA-Seq-Based Prognostic Model for Stage II–III CRC Patients

#### 2.1.1. Identification of Prognosis-Related Cell Subgroups in CRC scRNA-Seq Data

**Figure 1:**
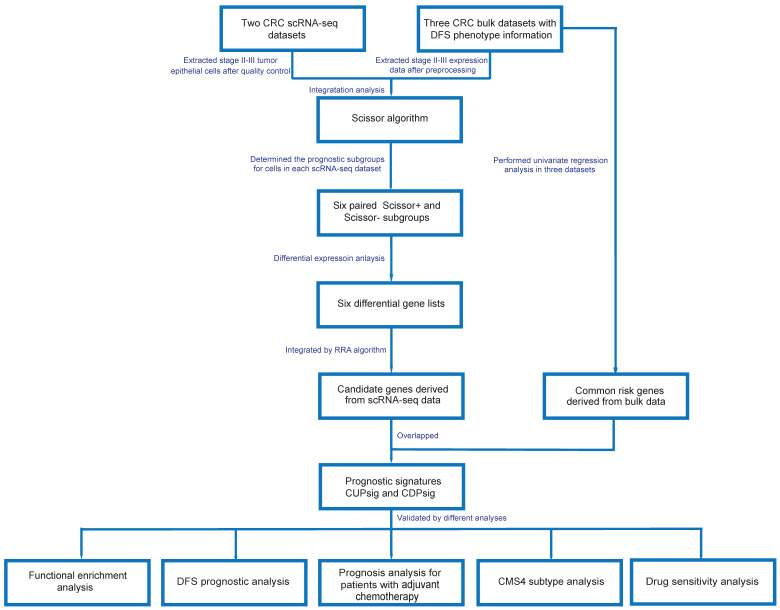
The flowchart for the identification of CRC prognosis-related gene signatures CUPsig and CDPsig.

**Figure 2:**
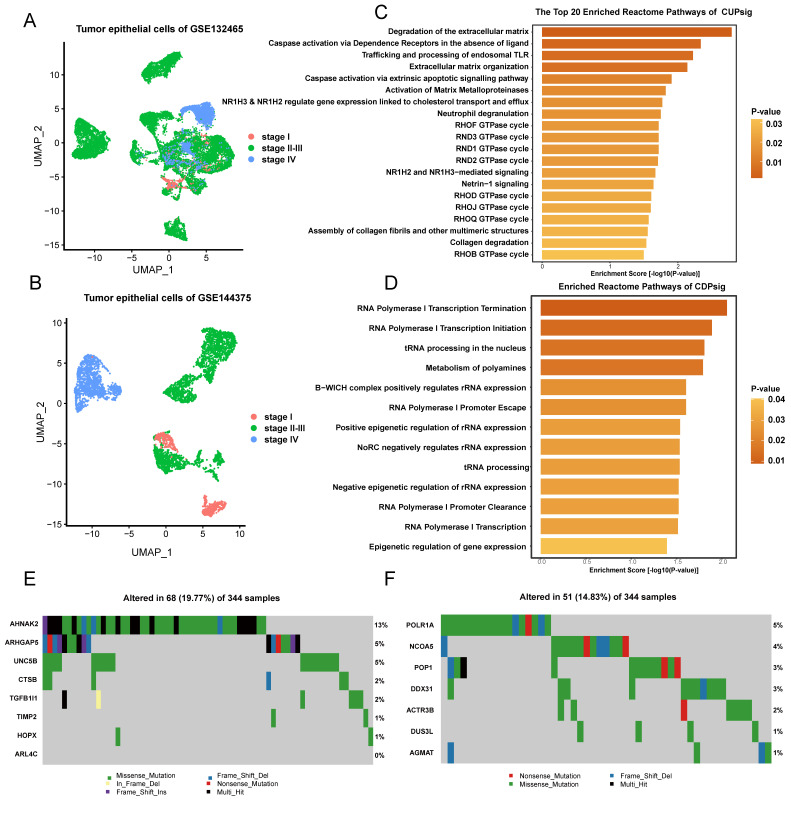
(**A**,**B**) UMAP plots of tumor epithelial cells in stage I, stage II–III, and stage IV of scRNA-seq datasets: (**A**) GSE132465, (**B**) GSE144735. (**C**,**D**) Results of Reactome enrichment analysis for CUPsig and CDPsig: (**C**) CUPsig, (**D**) CDPsig. (**E**,**F**) The mutation analysis of CUPsig and CDPsig in TCGA stage II–III CRC samples: (**E**) CUPsig, (**F**) CDPsig.

To eliminate the effects of non-disease cells in scRNA-seq data, we obtained 13,822 and 2778 stage II–III CRC tumor epithelial cells from GSE132465 and GSE144735, respectively (Table 1 and Figure 1 and Figure 2A,B). After that, based on the bulk detection expression data of 145, 461, and 130 stage II–III CRC patients with DFS survival information in GSE17538, GSE39582, and GSE37892, we used the Scissor algorithm to identify associations of CRC tumor epithelial cells in two scRNA-seq datasets with prognostic phenotypes of three bulk datasets and further to classify them into different Scissor+ and Scissor− cell subgroups. Within them, we considered that Scissor+ cell groups are related to poor prognosis of CRC, while Scissor− groups are associated with good prognosis. Prediction of 13,822 stage II–III tumor epithelial cells in GSE132465 based on GSE17538, GSE39582, and GSE37892, 1124 Scissor+ and 1410 Scissor− cells, 685 Scissor+ and 541 Scissor− cells, and 710 Scissor+ and 1353 Scissor− cells were identified. For 2778 stage II–III tumor epithelial cells of GSE144735, based on GSE17538, GSE39582, and GSE37892, 210 Scissor+ and 459 Scissor− cells, 275 Scissor+ and 154 Scissor− cells, and 381 Scissor+ and 401 Scissor− cells were recognized (Supplementary Table S1).

#### 2.1.2. Identification of CRC Prognosis-Related Genes

To further effectively identify prognosis-related gene signatures in stage II–III CRC patients, we performed differential expression analysis on the six pairs of Scissor+ and Scissor− cell subgroups obtained above, and the corresponding differential gene lists were generated (Supplementary Table S2). We then integrated them by the RRA algorithm based on the corresponding logFC values (Supplementary Table S3) [22]. Finally, 2584 up-regulated genes and 3200 down-regulated genes with RRA score less than 0.05 were selected as prognosis-related candidate genes of scRNA-seq data (Figure 1 and Supplementary Table S4). Meanwhile, we performed univariate Cox regression analysis in GSE17538, GSE37892, and GSE39582 and selected genes with *p* < 0.01 to constitute prognosis-related gene sets of bulk data (Figure 1). After intersection, we obtained eight and seven common up- and down-regulated risk genes, which were respectively marked with CUPsig and CDPsig. The CUPsig includes CTSB, TIMP2, AHNAK2, ARHGAP5, ARL4C, UNC5B, TGFB1I1, and HOPX, and CDPsig includes DUS3L, AGMAT, POP1, POLR1A, DDX31, ACTR3B, and NCOA5. Notably, we found that the majority of genes in the CUPsig and CDPsig were cancer related. CTSB, TIMP2, ARL4C, UNC5B, TGFB1I1, and HOPX, NCOA5, and AGMAT have been recorded in the DisGeNET database (https://www.disgenet.org/ (accessed on 11 November 2021)) as CRC known disease genes (Supplementary Table S5). For example, Bian et al. found that secretion of CTSB-encoded proteins was increased in the extracellular environment of CRC, thereby promoting cancer invasion and metastasis. In addition, Campo et al. found that high expression levels of CTSB in tumor epithelial cells of CRC patients were associated with significantly shorter survival of patients. Wang et al. showed that TIMP2 was a prognostic biomarker in CRC patients, and they confirmed that TIMP2 could directly affect cell invasion, migration, and angiogenesis in CRC patients and play an important role in prognosis. UNC5B was down-regulated in about 20% of CRC patients, and patients with low expression of UNC5B had a significantly higher recurrence rate after curative surgery. Similar to CTSB, CRC patients with high expression of ARL4C often showed poor survival rates and studies suggested that ARL4C could be used as a new therapeutic target to inhibit the proliferation and invasion of CRC cells. TGFB1I1 was considered a tumor suppressor gene in CRC and downregulated in CRC tissues and cell lines, its overexpression inhibited CRC cell proliferation, migration, invasion, and induced apoptosis. Meanwhile, overexpression of TGFB1I1 in CRC cells inhibited the TGF-β pathway and the progression of epithelial-mesenchymal transition (EMT). HOPX had also been shown to have tumor suppressor functions in various cancers including CRC, and HOPX might be involved in inhibiting CRC metastasis. Tian et al. found that ARHGAP5 expression was significantly increased in metastatic CRC tissues and negatively correlated with overall survival rates of patients, and ARHGAP5 promoted CRC cell EMT by negatively regulating RhoA activity. NCOA5 exhibited an oncogenic role in CRC and promoted CRC cell proliferation, migration, and invasion, while activating the PI3K/AKT signaling pathway. Zhu et al. mentioned that AGMAT could promote the progression of CRC by inducing chronic inflammation. To investigate the mutation status of these genes, we also downloaded mutation data of 344 stage II–III CRC patient samples in TCGA. Mutation analysis result showed that genes having the highest mutation frequencies in CUPsig and CDPsig were AHNAK2 and POLR1A, which respectively were 13% and 5% (Figure 2E,F).

Next, we performed Reactome enrichment analysis for CUPsig and CDPsig to explore what biological functions and related pathways they play. We found that CUPsig were significantly enriched in some pathways, such as RND3 GTPase cycle, RHOB GTPase cycle, Caspase activation via extrinsic apoptotic signaling pathway, and Activation of Matrix Metalloproteinases (Figure 2C and Supplementary Table S5). These pathways have been proved to be closely related to the occurrence or progression of cancer. Several studies have found that RND3 played an active role in human CRC invasion and metastasis, which was an independent prognostic marker of CRC [23,24,25]. In addition, studies on CRC patient biopsies have shown that RHOB was significantly under-expressed in CRC [26,27,28,29,30]. While Buttacavoli et al. found that Matrix Metalloproteinases were targeted in colon cancer and might serve as new biomarkers involved in immune response [31]. Most of genes of the CDPsig were enriched in tRNA-related pathways (Figure 2D). Additionally, normal tRNA metabolism is critical for maintaining the stability and function of tRNA molecules, but defects in certain tRNA biogenesis proteins contributed to a variety of human diseases, including cancer, neurological disorders, immunodeficiency, and diabetes [32].

### 2.2. Validation of CUPsig and CDPsig

#### 2.2.1. Prognostic Assessment of CUPsig and CDPsig

To further evaluate and verify whether the CUPsig and CDPsig have good performance in predicting the risk of tumor recurrence for stage II–III CRC patients, we performed survival analysis on 12 bulk data with DFS time, including TCGA, GSE17538, GSE39582, GSE37892, GSE38832, GSE14333, GSE31595, GSE29621, GSE92921, GSE161158, GSE17536, and GSE17537. Among them, the number of stage II–III CRC patients contained in these CRC validation datasets was 145, 461, 130, 74, 185, 37, 40, 59, 154, 111, 34, and 72, respectively (Table 1).

**Figure 3:**
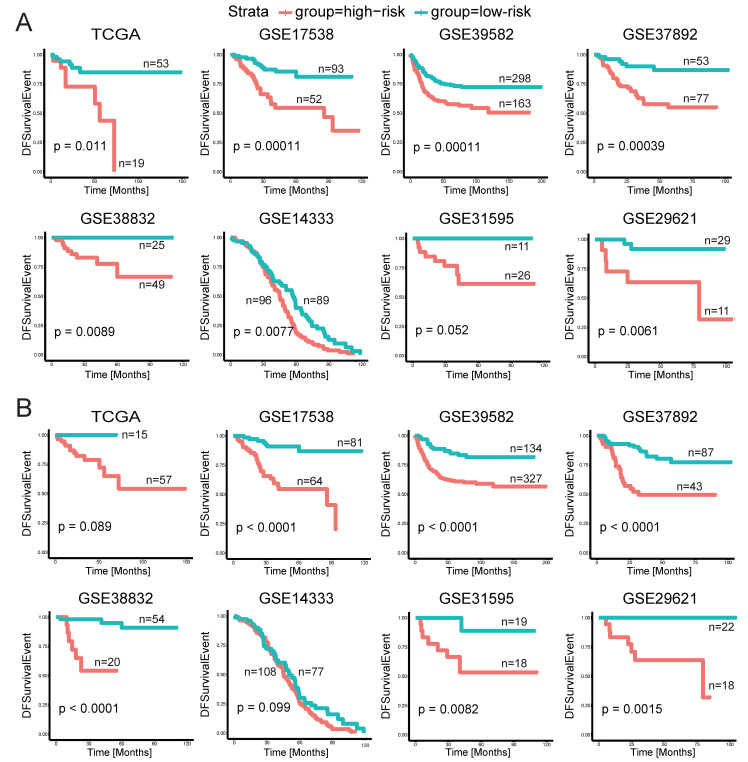
Kaplan–Meier survival curves of DFS between high-risk and low-risk groups in CRC validation datasets: (**A**) CUPsig, (**B**) CDPsig.

We found that CUPsig had good prognostic performance and this signature could effectively classify stage II–III CRC patients into high- and low-risk groups in 11 CRC validation datasets, including GSE17538 (*p* = 1.1 × 10^−4^), GSE39582 (*p* = 1.1 × 10^−4^), GSE37892 (*p* = 3.9 × 10^−4^), GSE38832 (*p* = 8.9 × 10^−3^), GSE14333 (*p* = 7.7 × 10^−3^), GSE29621 (*p* = 6.1 × 10^−3^), GSE92921 (*p* = 4.4 × 10^−4^), GSE161158 (*p* < 1 × 10^−3^), GSE17536 (*p* < 1 × 10^−3^), GSE17537 (*p* = 4.2 × 10^−3^), and TCGA (*p* = 0.011) (Figure 3A and Supplementary Figure S1A). Similarly, CDPsig performed well in classifying high- and low-risk patient groups with significantly different DFS in GSE17538, GSE39582, GSE37892, GSE38832, GSE31595, GSE29621, GSE92921, GSE161158, GSE17536, and GSE17537, log-rank *p*-values were <1 × 10^−3^, <1 × 10^−3^, <1 × 10^−3^, <1 × 10^−3^, 8.2 × 10^−3^, 1.5 × 10^−3^, 6.2 × 10^−3^, <1 × 10^−3^, <1 × 10^−3^, and 0.021, respectively. However, in GSE14333 and TCGA, the log-rank *p*-values of Kaplan–Meier survival analysis were 0.099 and 0.089 (Figure 3B and Supplementary Figure S1B). 

#### 2.2.2. Independent Prognostic Factors Assessment and Nomogram Construction

To further investigate whether CUPsig and CDPsig were independent clinical prognostic factors that were independent of other factors, such as age and sex, we performed univariate and multivariate Cox regression analysis on 11 CRC validation datasets that CUPsig successfully divided into high- and low-risk groups with significantly different DFS and the 10 CRC validation datasets that CDPsig successfully divided into high- and low-risk groups. The results of univariate analysis showed that CUPsig was significantly correlated with DFS in nine CRC validation datasets, including GSE17538 (HR = 0.272; *p* < 0.001), GSE39582 (HR = 0.524; *p* < 0.001), GE37892 (HR = 0.233; *p* = 0.001), GSE14333 (HR = 0.625; *p* = 0.008), GSE29621 ((HR = 0.137; *p* = 0.018), GSE92921 (HR = 0.089; *p* = 0.005), GSE161158 (HR = 0.286; *p* < 0.001), GSE17536 (HR = 0.235; *p* < 0.001), and TCGA (HR = 0.264; *p* = 0.018). Multivariate analysis was further carried out, and the results showed that CUPsig could be used as an independent prognostic factor for CRC patients in seven CRC validation datasets, including GSE17538 (HR = 0.628; *p* < 0.001), GSE39582 (HR = 0.519; *p* < 0.001), GE37892 (HR = 0.233; *p* = 0.001), GSE14333 (HR = 1.473; *p* = 0.033), GSE29621 (HR = 0.106; *p* = 0.014), GSE17536 (HR = 0.24; *p* < 0.001), and TCGA (HR = 0.263; *p* = 0.019) (Table 2). The univariate analysis results for CDPsig indicated that it was significantly correlated with DFS in six CRC validation datasets, containing GSE17538 (HR = 0.163; *p* < 0.001), GSE39582 (HR = 0.337; *p* < 0.001), GE37892 (HR = 0.283; *p* = 0.001), GSE92921 (HR = 0.145; *p* = 0.018), GSE161158 (HR = 0.177; *p* < 0.001), and GSE17536 (HR = 0.19; *p* < 0.001). Similarly, we then conducted multivariate analysis and found that CDPsig was an independent prognostic factor in GSE17538 (HR = 0.156; *p* < 0.001), GSE39582 (HR = 0.359; *p* < 0.001), GE37892 (HR = 0.272; *p* = 0.001), GSE161158 (HR = 0.178; *p* < 0.001), and GSE17536 (HR = 0.189; *p* < 0.001) (Table 3).

**Figure 4:**
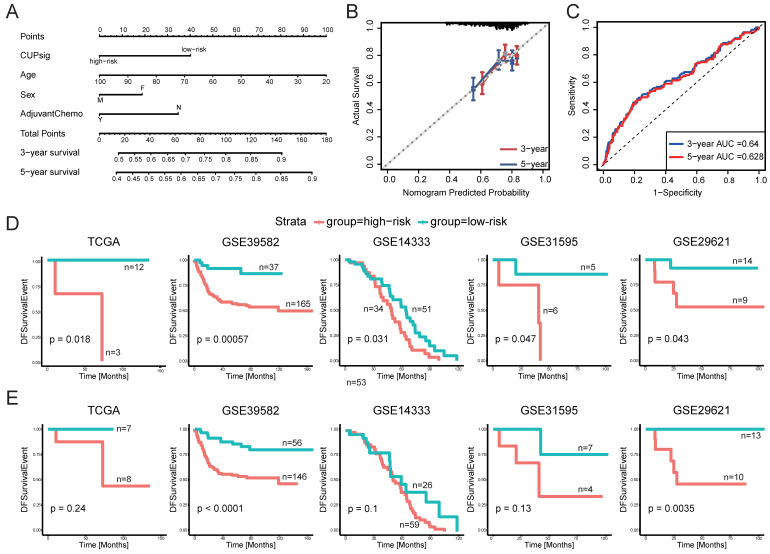
(**A**–**C**) Nomogram, calibration curve, and the AUC of nomogram model based on ROC curve used to predict the disease-free survival time of patients with stage II–III CRC in GSE39582: (**A**) nomogram, (**B**) calibration curve, (**C**) ROC curve. (**D**,**E**) Evaluation of the predictive power of CUPsig and CDPsig in stage II–III CRC patients receiving adjuvant chemotherapy. Kaplan–Meier survival curves of DFS in high-risk and low-risk patients with stage II–III CRC in the five drug-treated validation datasets (TCGA-5FU-based, GSE39582-5FU-based, GSE14333-5FU-based, GSE29621-5FU-based, and GSE31595-drug-unknown), while other seven datasets without adjuvant chemotherapy information were not used: (**D**) CUPsig, (**E**) CDPsig.

After multivariate Cox analysis of clinical parameters, CUPsig, sex, age, and adjuvant chemotherapy were still strong independent factors in predicting DFS in GSE39582. Based on this, a nomogram was developed by integrating CUPsig, sex, age, and adjuvant chemotherapy to predict DFS (Figure 4A). The usefulness of the comprehensive nomogram was also confirmed in the time-dependent ROC analysis, with 3- and 5-year areas under the curve (AUC) of 0.64 and 0.628 for predicting DFS, respectively (Figure 4B). In addition, the calibration curve showed the high accuracy of the comprehensive nomogram model to predict DFS (Figure 4C).

### 2.3. Predictive Power of CUPsig and CDPsig in Patients Receiving Adjuvant Chemotherapy

Surgery is the mainstay of treatment for CRC, and adjuvant chemotherapy is clinically recommended in high-risk stage II and III CRC patients to reduce the risk of local recurrence and prolong DFS for them [33,34]. Therefore, we further investigated the predictive powers of CUPsig and CDPsig for patients receiving adjuvant chemotherapy in five drug-treated validation datasets containing adjuvant chemotherapy information, including GSE39582 (*n* = 202), GSE14333 (*n* = 85), GSE31595 (*n* = 11), TCGA (*n* = 15), and GSE29621 (*n* = 23). The clinical information of the first four datasets showed that patients in them were treated with 5-FU based adjuvant chemotherapy (Table 1), while the last one had no drug information. Therefore, we used the combined name with the GSE accession number and the adjuvant chemotherapy drug to label them. We found that CUPsig successfully classified stage II–III patients receiving adjuvant chemotherapy into high- and low-risk groups with markedly different DFS in five drug-treated validation datasets: GSE39582-5FU-based (*p* = 5.7 × 10^−4^), GSE14333-5FU-based (*p* = 0.031), GSE29621-5FU-based (*p* = 0.043), TCGA-5FU-based (*p* = 0.018), and GSE31595-drug-unknown (*p* = 0.047) (Figure 4D). In addition, CDPsig had good performance in stratifying stage II–III CRC patients receiving adjuvant chemotherapy into high- and low-risk groups in GSE29621-5FU-based (*p* = 3.5 × 10^−3^) and GSE39582-5FU-based (*p* < 1 × 10^−3^) (Figure 4E). The results showed that CUPsig and CDPsig established by our method had good ability to predict DFS in stage II–III CRC patients who received adjuvant chemotherapy.

### 2.4. Predictive Power of CUPsig and CDPsig in CMS4 Subtype Patients

**Figure 5:**
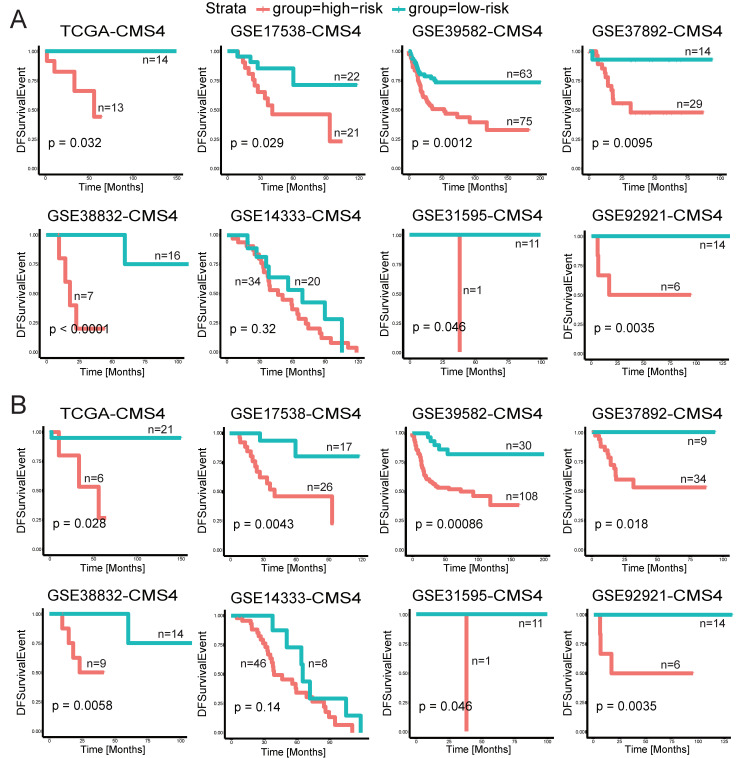
Evaluation of the predictive power of CUPsig and CDPsig in CMS4 subtype patients with stage II–III CRC. Kaplan–Meier survival curves of DFS in high-risk and low-risk patients with stage II–III CRC in validation datasets: (**A**) CUPsig, (**B**) CDPsig.

The consensus molecular subtypes (CMS) proposed by Guinney et al. is of great significance for the clinical diagnosis and prognosis of CRC [35]. Previous clinical analyses had shown that adjuvant chemotherapy had poor efficacy on CMS4 subtype cells, and patients with CMS4 subtype had the worst five-year overall survival (62%) and relapse-free survival (60%) [36]. According to this assumption, we further evaluated the prognostic performance of CUPsig and CDPsig in CMS4 subtype patients. Before this step, we performed the CMS subtype prediction in patients of 12 CRC validation datasets. In total, 1371 CMS subtype patients were obtained (Supplementary Table S6). We then selected 455 CMS4 subtype patients with stage II–III CRC (27 from TCGA, 43 from GSE17538, 138 from GSE39582, 43 from GSE37892, 23 from GSE38832, 54 from GSE14333, 12 from GSE31595, 13 from GSE29621, 20 from GSE92921, 41 from GSE161158, 34 from GSE17536, and 7 from GSE17537) for the following analysis (Table 4). Through survival analysis results, we found that CUPsig could divide CMS4 subtype patients from multiple CRC validation datasets into high- and low-risk groups with significantly different DFS, including TCGA (*p* = 0.032), GSE17538 (*p* = 0.029), GSE39582 (*p* = 1.2 × 10^−3^), GSE37892 (*p* = 9.5 × 10^−3^), GSE38832 (*p* < 1 × 10^−3^), GSE31595 (*p* = 0.046), GSE92921 (*p* = 3.5 × 10^−3^), GSE161158 (*p* = 0.022), and GSE17536 (*p* = 0.02) (Figure 5A and Supplementary Figure S1C). Similarly, CDPsig could successfully stratify CMS4 subtype patients into high- and low-risk groups in nine CRC validation datasets, containing TCGA (*p* = 0.028), GSE17538 (*p* = 4.3 × 10^−3^), GSE39582 (*p* = 8.6 × 10^−4^), GSE37892 (*p* = 0.018), GSE38832 (*p* = 5.8 × 10^−3^), GSE31595 (*p* = 0.046), GSE92921 (*p* = 3.5 × 10^−3^), GSE161158 (*p* = 1.5 × 10^−3^), and GSE17536 (*p* = 2.1 × 10^−3^) (Figure 5B and Supplementary Figure S1D). These results indicated that CUPsig and CDPsig still have certain power for predicting DFS in CMS4 subtype patients, which could provide references for further research in CMS4 subtype patients with stage II–III CRC.

### 2.5. The Relationship between CUPsig and CDPsig Expression and Drug Sensitivity

**Figure 6:**
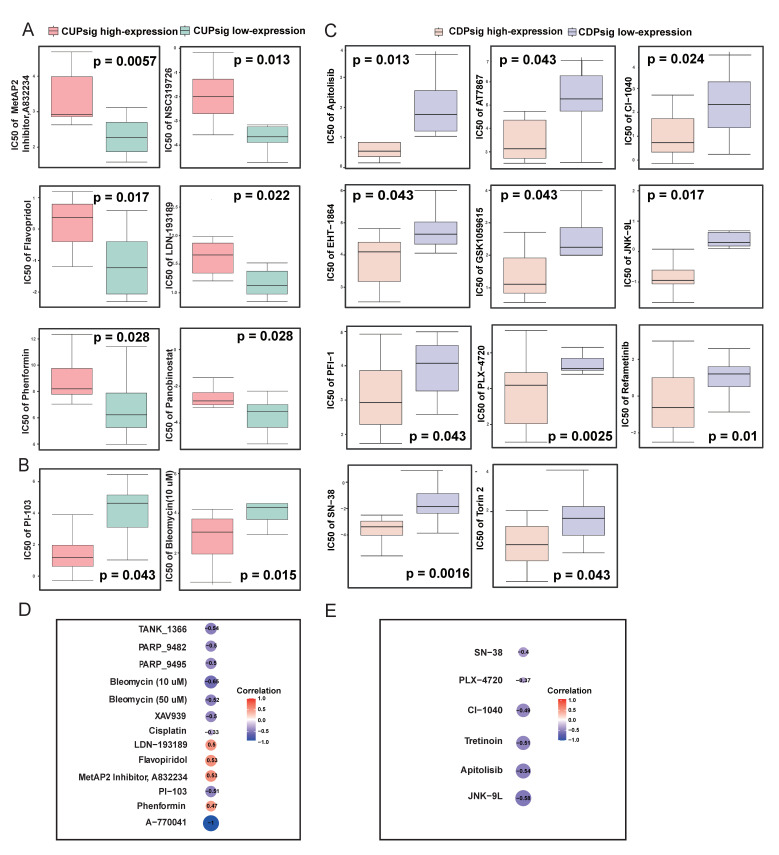
The relationships between CUPsig and CDPsig expressions and drug sensitivities: (**A**,**B**) Differences in IC50 values of CRC cell lines treated with eight drug components between CUPsig high- and low-expression groups; (**C**) differences in IC50 values of CRC cell lines treated with 11 drug components between CDPsig high- and low-expression groups; (**D**,**E**) correlations between CUPsig and CDPsig expression levels and drug sensitivity (IC50). A positive (or negative) correlation means that the CRC cell line with high expression of CUPsig and CDPsig was resistant (or sensitive) to the drug: (**D**) CUPsig, (**E**) CDPsig.

Based on expression profile data of 20 CRC cell lines from the Cancer Cell Line Encyclopedia (CCLE, https://sites.broadinstitute.org/ccle/ (accessed on 13 June 2022)) and drug sensitivity (IC50) data from Genomics of Drug Sensitivity in Cancer (GDSC, https://www.cancerrxgene.org/ (accessed on 6 September 2021)), we performed the drug sensitivity analysis and found that the IC50 values of six drugs in CUPsig low-expression group were significantly lower than CUPsig high-expression group, which contained MetAP2 Inhibitor (*p* = 5.7 × 10^−3^), NSC319726 (*p* = 0.013), Flavopiridol (*p* = 0.017), LDN-193189 (*p* = 0.022), Phenformin (*p* = 0.028), and Panobinostat (*p* = 0.028). This result indicated that CRC cell lines with low expression of CUPsig were more sensitive to the above six drugs (Figure 6A) [37,38]. Meanwhile, IC50 values of two drugs (PI-103 and Bleomycin (10 μM)) in the CUPsig high-expression group were significantly lower than those in the CUPsig low-expression group (*p* = 0.043 and 0.015), illustrating that CRC cell lines with high expression of CUPsig were more sensitive to them (Figure 6B). In addition, analysis results for CDPsig showed that CRC cell lines with high expression of CDPsig were more sensitive to Apitolisib (*p* = 0.013), AT7867 (*p* = 0.043), CI-1040 (*p* = 0.024), EHT-1864 (*p* = 0.043), GSK1059615 (*p* = 0.043), JNK-9L (*p* = 0.017), PFI-1 (*p* = 0.043), PLX-47209 (*p* = 2.5 × 10^−3^), Refametinib (*p* = 0.01), SN-38 (*p* = 1.6 × 10^−3^), and Torin 2 (*p* = 0.043) (Figure 6C).

Next, drug sensitivity analysis was performed. We found that CUPsig and CDPsig expressions were significantly associated with sensitivities in CRC cell lines to multiple drugs (*p* < 0.05). For instance, higher expression levels of CUPsig were associated with increased resistance of CRC cell lines to Flavopiridol, MetAP2 Inhibitor, LDN-193189, and Phenformin; however, it resulted in increased sensitivity of cell lines to TANK_1366, PARP_9482, XAV939, Cisplatin, PARP_9495, PI-103, Bleomycin (50 μM), Bleomycin (10 μM), and A-770041 (Figure 6D). What is more, CRC cell lines with higher CDPsig expression were more sensitive to SN−38, PLX−4720, CI−1040, Tretinoin, Apitolisib, and JNK−9L (Figure 6E).

## 3. Discussion

Effectively identifying the prognosis and adjuvant chemotherapy-related gene signatures is the key to guiding the prognostic treatment for stage II–III CRC patients who need adjuvant chemotherapy in clinical application. Therefore, in this study, we developed a scRNA-seq-based prognostic signature identification method by integrating single-cell and bulk data to identify two novel prognosis-related signatures (CUPsig and CDPsig). Through comprehensive evaluation and verification, we found that CUPsig and CDPsig had broad and good prognostic efficacy, suggesting that these two signatures may be potential predictive biomarkers for adjuvant-treated stage II–III CRC.

Due to the samples in bulk data detected by traditional RNA-seq or microarray, they generally are mixed with a large number of non-cancer samples, such as tumor microenvironment or adjacent tissue samples. In addition, the gene biomarker expression values detected by these techniques are usually averaged across all samples. The scRNA-seq technology improves the limitations of the above traditional detection methods to a certain extent, but this technology cannot effectively identify the associations between cells and the prognosis of cancer patients at the cellular level. Therefore, we systematically integrated scRNA-seq and bulk data by our method to identify and establish two more accurate prognostic signatures, a CUPsig and a CDPsig. Through extensive text mining and data searching, we found that CTSB, TIMP2, ARHGAP5, ARL4C, UNC5B, TGFB1I1, and HOPX in CUPsig have been confirmed as CRC-related genes. Among these genes NCOA5, AGMAT, POP1, ACTR3B, DDX31, POLR1A, and DUS3L in CDPsig, NCOA5 and AGMAT have been confirmed to be related to CRC. There was a study found that POP1 was differentially expressed in CRC and an up-regulated trend in CRC tissues, which could be used as a prognostic factor in CRC [39]. Yu et al. found for the first time that ACTR3B expression was significantly increased in CRC tissues compared with matched normal tissues and confirmed that AC009022.1 promoted ACTR3B expression by inhibiting miR-497-5p and enhanced CRC cell proliferation, migration, and invasion [40]. Furthermore, we analyzed our prognostic signatures in stage II–III adjuvant radiotherapy patients with GSE14333 (*n* = 22), where CDPsig (*p* = 1.9 × 10^−3^) successfully divided adjuvant radiotherapy patients into high- and low-risk groups (Supplementary Figure S2A,B). We also compared the expression differences of CUPsig and CDPsig among the four CMS subtypes, respectively, and found that CUPsig was significantly overexpressed in the CMS4 subtype, while CDPsig was generally highly expressed in the CMS2 subtype (Supplementary Figure S2C,D). Considering that CRC is essentially composed of colon and rectum cancers (CC and RC), we re-evaluated and verified whether CUPsig and CDPsig can effectively predict the benefits of adjuvant chemotherapy during the adjuvant chemotherapy analysis. In all CRC validation datasets, only GSE14333 has phenotypes; therefore, we divided CRC patient samples (*n* = 85) in GSE14333 into CC (*n* = 77) and RC (*n* = 8) and reperformed the prognosis analysis based on them. The log-rank *p*-values of CUPsig and CDPsig in CC and RC are 0.16 and 0.98, and 0.26 and 0.13, respectively. No significant results were found, which may be caused by the smaller sample size of CC and RC after the classification. Moreover, through the DrugBank database (https://go.drugbank.com/ (accessed on 5 October 2022)), we searched known drug targets for genes in CUPsig and CDPsig. We found CTSB in CUPsig was a target of Trastuzumab deruxtecan, which had been approved for certain types of metastatic or unresectable breast cancer [41,42]. The result indicated that the gene might be a potential drug target and provide a direction for the targeted therapy of CRC.

In summary, we developed a bioinformatics approach to identify and establish an eight-gene prognostic signature CUPsig and a seven-gene prognostic signature CDPsig. Our findings indicated that the two prognostic-related signatures could be used as novel and potential prognostic factors for prognostic diagnosis of stage II–III CRC patients, which could provide potential and effective prognostic tools for the optimization of treatment decisions for stage II–III CRC patients who received adjuvant chemotherapy. 

## 4. Materials and Methods

### 4.1. Data Collection and Preprocessing

#### 4.1.1. scRNA-Seq Datasets

We downloaded current available CRC-associated scRNA-seq datasets from Gene Expression Omnibus (GEO) and received GSE132465 (https://www.ncbi.nlm.nih.gov/geo/query/acc.cgi?acc=GSE132465 (accessed on 24 March 2021)) and GSE144735 (https://www.ncbi.nlm.nih.gov/geo/query/acc.cgi?acc=GSE144735 (accessed on 24 March 2021)), which were separately detected by Illumina HiSeq 4000 (GPL20301) and Illumina NovaSeq 6000 (GPL24676) platforms. These two datasets, respectively, include 63,689 and 27,414 cells of 23 and 6 CRC patients, and 33,694 genes were obtained after scRNA-seq data and were aligned to the human reference genome (GRCh38) (Table 1).

Next, we performed quality control on each scRNA-seq data to filter low-quality cells based on the following criteria: cells with unique molecular identifier (UMI) counts >1000, gene counts between 200 and 6000, and mitochondrial gene expression below 20% in UMI counts. Similar quality control was performed on genes; protein-coding genes were extracted, and genes expressed in at least 3 cells were retained. Finally, 19 and 4 stage II–III patients with 13,822 and 2778 tumor epithelial cells were obtained. After that, 16,845 and 16,513 protein-coding genes in GSE132465 and GSE144735 were remained. The above step was completed by the R package “Seurat” [43].

#### 4.1.2. Bulk Datasets for Validation

Totally, 1753 frozen tissue samples from 9 stage I-IV CRC bulk datasets, including GSE17538, GSE39582, GSE37892, GSE38832, GSE14333, GSE31595, GSE29621, GSE92921, and GSE161158 were collected from GEO, all of them were detected by the Affymetrix Human Genome U133 Plus 2.0 Array (GPL570) platform (Table 1). Among them, 145, 461, 130, 74, 185, 37, 40, 59, and 154 stage II–III CRC patients were received. GSE17536 and GSE17537 belonging to the GSE17538 contain 111 and 34 stage II–III CRC patients, respectively. Normalization was then performed to these validation datasets by R package “fRMA” [44]. 

An additional CRC bulk dataset for validation (Table 1) was obtained from TCGA (UCSC Xena; https://xenabrowser.net/(accessed on 18 November 2021)), which was sequenced by the Illumina platform. In TCGA dataset, 72 out of 234 stage II–III CRC patients were collected and raw counts for each gene were log2(count + 1) transformed. In all CRC validation datasets, drug-treated validation datasets, including GSE39582, GSE14333, GSE31595, TCGA, and GSE29621, were used for the adjuvant chemotherapy analysis, while the remaining datasets lacking specific adjuvant chemotherapy and drug information were deleted.

### 4.2. Integration of scRNA-Seq Datasets and Bulk Datasets to Identify CRC Prognostic Associated Signatures

#### 4.2.1. Identification of Prognostic Associated Cells

The three microarray datasets of GSE39582, GSE17538, and GSE37892 have relatively comprehensive stage II–III CRC expression data and DFS prognostic information. We integrated them with scRNA-seq datasets of GSE132465 and GSE144735 by the Scissor algorithm to identify cell subgroups that are most highly associated with the stage II–III CRC patients’ prognostic phenotypes. 

After that, Scissor+, Scissor−, and the prognostic-unrelated background cell subgroups corresponding to GSE132465 and GSE144735 were identified.

#### 4.2.2. Selection of Differential Genes between Scissor+ and Scissor− Cell Subgroups

Subsequently, we calculated the differential genes for Scissor+ and Scissor− cell subgroups using the R package “limma” and obtained six differential expression gene lists. Then, the RobustRankAggreg (RRA) algorithm was used to integrate them according to the logFC values; after that, we obtained a list of up-regulated differential genes and a list of down-regulated differential genes. The common up-regulated or down-regulated genes with RRA score less than 0.05 were regarded as candidate genes.

#### 4.2.3. Identification and Construction of Prognostic Associated Signatures

Meanwhile, we separately performed univariate Cox regression in GSE39582, GSE17538, and GSE37892 stage II–III expression data to obtain risk genes that were significantly associated with the DFS of CRC patients (*p* < 0.01). The overlap genes of common risk genes of the three datasets and candidate genes were used to construct a CUPsig and a CDPsig. We downloaded TCGA mutation data from UCSC (https://xenabrowser.net/(accessed on 18 November 2021)), after extracting 344 stage II–III samples. The R package “maftools” was used to perform mutation analysis [45]. Then, we performed Reactome enrichment analysis for CUPsig and CDPsig by using the R package “ReactomePA” (*p* < 0.05) [46].

### 4.3. CRC Prognostic Signatures Validation

#### 4.3.1. Evaluation of CUPsig and CDPsig in the Validation Cohorts

For the bulk CRC validation datasets TCGA, GSE17538, GSE39582, GSE37892, GSE38832, GSE14333, GSE31595, GSE29621, GSE92921, GSE161158, GSE17536, and GSE17537, the R package “pROC” was used to choose the best risk score thresholds to classify high- and low-risk groups [47]. The risk score formula was used to calculate the risk score for each stage II–III CRC patient based on a linear combination of expression values weighted by regression coefficients from univariate Cox regression analysis as shown below:

$$\mathrm{Risk}\;\mathrm{score}=\overset{n}{\underset{i=1}{\sum }}r_{i}Exp\left(i\right)$$

where *r_i_* is the Cox regression coefficient for gene *i* in CUPsig or CDPsig, *n* is the number of genes included in CUPsig or CDPsig, and *Exp*(*i*) is the expression value of gene *i* in the corresponding patient [48]. 

For all the above bulk validation datasets, Kaplan–Meier analysis and log-rank test were performed to assess the differences in DFS between high- and low-risk groups using R package “survival”.

To verify whether CUPsig and CDPsig can act as independent prognostic factors, univariate and multivariate Cox regression analysis were performed on CUPsig and CDPsig and several clinical parameters (age, sex, and adjuvant chemotherapy) by the R package “survival”. After that, a nomogram was constructed to predict DFS for patients with stage II–III CRC. Calibration curve and time-dependent ROC curve analysis were used to validate the accuracy of the nomogram model for predicting 3- and 5-year DFS of patients with stage II–III CRC.

#### 4.3.2. Adjuvant Chemotherapy Analysis

For five drug-treated validation datasets, we also divided high- and low-risk groups according to the risk score thresholds and performed survival analysis.

#### 4.3.3. CMS4 Subtype Analysis

We performed CMS subtype identification on patients in all CRC validation datasets by using the R package “CMScaller”; then, survival analysis was performed on patients with stage II–III CMS4 subtype CRC [49].

#### 4.3.4. Drug Sensitivity Analysis

We investigated the relations between CUPsig and CDPsig expressions and drug sensitivities. We downloaded the expression profile data of 20 CRC cell lines from the Cancer Cell Line Encyclopedia. The corresponding medication and IC50 information were downloaded from Genomics of Drug Sensitivity in Cancer. CRC cell lines were, respectively, divided into high- and low-expression groups according to the median expression value of CUPsig and CDPsig, and we compared the differences in IC50 values of compounds between these two groups by the Wilcoxon rank sum test. Next, we used Spearman correlation coefficients to assess relationships between CUPsig and CDPsig expression levels and drug sensitivity of 345 compounds, respectively (*p* < 0.05 was considered significantly related).

## Figures and Tables

**Table 1:** Summary of scRNA-seq datasets and validation datasets.

Datasets	Sample Types	Cells/ Patients	Stage II–III Tumor Epithelial Cells/Patients	Adjuvant Chemotherapy Patients	Adjuvant Chemotherapy Drugs	Platforms	PMID
scRNA-seq datasets							
GSE132465	Colorectal cancer	63,689 (23)	13,822 (19)			GPL20301	32451460 [13]
GSE144735	Colorectal cancer	27,414 (6)	2778 (4)			GPL24676	32451460
Validation datasets							
GSE17538	Colon cancer	200	145			GPL570	19914252 [14]
GSE37892	Colon cancer	130	130			GPL570	22917480 [15]
GSE38832	Colon cancer	122	74			GPL570	25320007 [16]
GSE92921	Colon cancer	59	59			GPL570	
GSE161158	Colorectal cancer	250	154			GPL570	34114372 [17]
GSE17536	Colon cancer	145	111			GPL570	19914252
GSE17537	Colon cancer	55	34			GPL570	19914252
TCGA	Colorectal cancer	234	72	15	Oxaliplatin/C-apecitabine/ Fluorouracil/5-FU/FolFox/ Calcium Foliatum, fluorouracilu, oxaliplatinum, dexamethassone/Xelo-da	Illumina HiSeq 2000	
GSE39582	Colon cancer	566	461	202	fluorouracil and folinic acid	GPL570	23700391 [18]
GSE14333	Colorectal cancer	226	185	85	5-fluouracil/ capecitabine/ 5-fluouracil and oxalplatin	GPL570	19996206 [19]
GSE29621	Colon cancer	65	40	23	5-fluouracil	GPL570	22362069 [20]
GSE31595	Colon cancer	37	37	11	Drug-unknown	GPL570	22710688 [21]

**Table 2:** Univariate and multivariate Cox regression analysis results of CUPsig in validation datasets. * *p* < 0.05.

Risk Factor	Univariate Analysis			Multivariate Analysis		
	HR	95% CI	*p* Value	HR	95% CI	*p* Value
TCGA						
CUPsig (high vs. low)	0.264	0.088–0.793	0.018 *	0.263	0.086–0.805	0.019 *
Age	1.01	0.969–1.053	0.631	1.001	0.953–1.015	0.979
Sex	5.18	1.127–23.821	0.035 *	5.068	1.031–24.907	0.046 *
GSE17538						
CUPsig (high vs. low)	0.272	0.134–0.551	<0.001 *	0.268	0.132–0.545	<0.001 *
Age	0.991	0.967–1.016	0.481	0.989	0.964–1.015	0.397
Sex	1.011	0.516–1.982	0.975	0.866	0.427–1.754	0.689
GSE39582						
CUPsig (high vs. low)	0.524	0.376–0.731	<0.001 *	0.519	0.37–0729	<0.001 *
Age	1.007	0.994–1.021	0.284	1.017	1.002–1.032	0.022 *
Sex	1.316	0.935–1.854	0.116	1.436	1.015–2.031	0.041 *
Adjuvant-Chemo (Y vs. N)	1.582	1.132–2.211	0.007 *	1.598	1.122–2.274	0.009 *
GSE37892						
CUPsig (high vs. low)	0.233	0.097–0.56	0.001 *	0.233	0.097–0.56	0.001 *
Age	0.991	0.967–1.016	0.49	0.994	0.971–1.018	0.643
Sex	1.15	0.599–2.206	0.675	1.203	0.624–2.319	0.58
GSE38832						
CUPsig (high vs. low)			0.998			
GSE14333						
CUPsig (high vs. low)	0.625	0.441–0.886	0.008 *	1.473	1.032–2.103	0.033 *
Age	1.017	1.003–1.031	0.019 *	1.012	0.998–1.027	0.092
Sex	0.978	0.695–1.374	0.897	1	0.71–1.409	0.999
Adjuvant-Chemo (Y vs. N)	0.696	0.494–0.98	0.038 *	0.805	0.562–1.152	0.235
GSE29621						
CUPsig (high vs. low)	0.137	0.026–0.715	0.018 *	0.106	0.018–0.631	0.014 *
Age	1.628	0.311–8.531	0.564	1.621	0.314–8.382	0.564
Sex	1.083	0.242–4.849	0.917	0.489	0.093–2.582	0.399
GSE92921						
CUPsig (high vs. low)	0.089	0.016–0.488	0.005 *			
GSE161158						
CUPsig (high vs. low)	0.286	0.151–0.542	<0.001 *	0.285	0.15–0.54	<0.001 *
Age	0.286	0.151–0.542	<0.001 *	0.992	0.969–1.015	0.473
GSE17536						
CUPsig (high vs. low)	0.235	0.111–0.5	<0.001 *	0.24	0.112–0.514	<0.001 *
Age	0.986	0.963–1.01	0.25	0.99	0.963–1.018	0.471
Sex	1.138	0.562–2.304	0.72	0.873	0.409–1.863	0.725
GSE17537						
CUPsig (high vs. low)			0.999			0.999
Age	1.063	0.952–1.186	0.28	1.382	1.171–1.631	<0.001 *
Sex	0.569	0.051–6.33	0.65	261.793	11.193–6123.202	<0.001 *

**Table 3:** Univariate and multivariate Cox regression analysis results of CDPsig in validation datasets. * *p* < 0.05.

Risk Factor	Univariate Analysis			Multivariate Analysis		
	HR	95% CI	*p* Value	HR	95% CI	*p* Value
GSE17538						
CDPsig (high vs. low)	0.163	0.071–0.376	<0.001 *	0.156	0.067–0.362	<0.001 *
Age	0.991	0.967–1.016	0.481	0.983	0.956–1.011	0.235
Sex	0.163	0.071–0.376	<0.001 *	0.96	0.485–1.899	0.906
GSE39582						
CDPsig (high vs. low)	0.337	0.21–0.542	<0.001 *	0.359	0.223–0.578	<0.001 *
Age	1.007	0.994–1.021	0.284	1.012	0.998–1.026	0.101
Sex	1.943	1.388–2.721	<0.001 *	1.565	1.037–2.36	0.033 *
Adjuvant-Chemo (Y vs. N)	1.582	1.132–2.211	0.007	1.263	0.826–1.933	0.282
GSE37892						
CDPsig (high vs. low)	0.283	0.147–0.544	<0.001 *	0.272	0.14–0.526	<0.001 *
Age	0.991	0.967–1.016	0.49	0.993	0.971–1.015	0.532
Sex	1.15	0.599–2.206	0.675	1.335	0.689–2.589	0.392
GSE38832						
CDPsig (high vs. low)	0.073	0.015–0.371	0.002 *			
GSE31595						
CDPsig (high vs. low)	0.099	0.012–0.814	0.031 *	0.058	0.006–0.573	0.015 *
Age	1.026	0.955–1.103	0.48	1.09	0.982–1.209	0.105
Sex	1.008	0.239–4.256	0.992	0.924	0.201–4.246	0.919
Adjuvant-Chemo (Y vs. N)	2.138	0.532–8.602	0.285	4.012	0.708–22.742	0.117
GSE29621						
CDPsig (high vs. low)			0.999			0.999
Sex			0.999	1.743	0.379–8.019	0.475
Adjuvant-Chemo (Y vs. N)	1.628	0.311–8.531	0.564	1.58	0.287–8.714	0.599
GSE92921						
CDPsig (high vs. low)	0.145	0.029–0.721	0.018 *			
GSE161158						
CDPsig (high vs. low)	0.177	0.091–0.345	<0.001 *	0.178	0.092–0.346	<0.001 *
Age	0.993	0.97–1.015	0.52	0.994	0.969–1.019	0.612
GSE17536						
CDPsig (high vs. low)	0.19	0.089–0.407	<0.001 *	0.189	0.088–0.405	<0.001 *
Age	0.986	0.963–1.01	0.25	0.988	0.962–1.016	0.401
Sex	0.19	0.089–0.407	<0.001 *	1.211	0.579–2.533	0.611
GSE17537						
CDPsig (high vs. low)			0.999			0.999
Age	1.063	0.926–1.255	0.279	1.078	0.926–1.255	0.333
Sex			0.999	1.522	0.090–25.849	0.771

**Table 4:** Summary of CMS subtype patients with stage II–III CRC.

	CMS1	CMS2	CMS3	CMS4	TOTAL
TCGA	12	17	9	27	65
GSE17538	32	42	18	43	135
GSE39582	74	134	69	138	415
GSE37892	11	40	22	43	116
GSE38832	15	23	11	23	72
GSE14333	35	49	27	54	165
GSE31595	7	5	11	12	35
GSE29621	10	7	7	13	37
GSE92921	5	16	10	20	51
GSE161158	37	43	22	41	143
GSE17536	25	31	13	34	103
GSE17537	9	10	7	7	34

## Data Availability

All data in this study are publicly available data, and the GEO data numbers and other data download addresses are provided in the text.

## Supplementary Materials

The following supporting information can be downloaded at: https://www.mdpi.com/article/10.3390/ijms232012460/s1. References [50,51,52,53,54,55,56,57,58,59,60] are cited in the Supplementary Materials.
