# Supplementary material for: Identification of Prognostic Gene Signatures by Developing a scRNA-Seq-Based Integration Approach to Predict Recurrence and Chemotherapy Benefit in Stage II–III Colorectal Cancer
Source: Int J Mol Sci. 2022 Oct 18;23(20):12460. doi: 10.3390/ijms232012460 (PMC9604003; doi:10.3390/ijms232012460)
Supplement: Supplementary file 1 [file ijms-23-12460-s001.zip › Supplementary Figure Legends.pdf]

Supplementary Figure Legends:

Supplementary Figure S1: (A-B) Kaplan-Meier survival curves of DFS between high-risk and low-risk groups in CRC validation datasets. (A) CUPsig, (B) CDPsig. (C-D) Evaluation of the predictive power of CUPsig and CDPSig in CMS4 subtype patients with stage II-III CRC. Kaplan-Meier survival curves of DFS in high-risk and low-risk patients with stage II-III CRC in validation datasets. (C) CUPsig, (D) CDPsig.

Supplementary Figure S2: (A-B) Evaluation of the predictive ability of CUPsig and CDPSig in patients with stage II-III CRC treated with adjuvant radiotherapy. Kaplan-Meier survival curves of DFS in high-risk and low-risk patients with stage II-III CRC in GSE14333 (n=22). (A) CUPsig, (B) CDPsig. (C-D) The differences in the expression of prognostic signatures among the four CMS subtypes in the validation datasets. (C) CUPsig, (D) CDPsig.
