# Supplementary material for: Identification of Prognostic Gene Signatures by Developing a scRNA-Seq-Based Integration Approach to Predict Recurrence and Chemotherapy Benefit in Stage II–III Colorectal Cancer
Source: Int J Mol Sci. 2022 Oct 18;23(20):12460. doi: 10.3390/ijms232012460 (PMC9604003; doi:10.3390/ijms232012460)
Supplement: Supplementary file 1 [file ijms-23-12460-s001.zip › Supplementary Table S5.pdf]

Supplementary Table S5. Reactome pathway enrichment analysis results of CUPsig and CDPSig

Signatures	Reactome Pathways	P-values
**CUPsig**		
**CTSB [50, 51]**	Degradation of the extracellular matrix	0.002
	Caspase activation via Dependence Receptors in the absence of ligand	0.005
	Trafficking and processing of endosomal TLR	0.006
**TIMP2 [52]**	Extracellular matrix organization	0.007
	Caspase activation via extrinsic apoptotic signalling pathway	0.012
	Activation of Matrix Metalloproteinases	0.015
**AHNAK2**	NR1H3 & NR1H2 regulate gene expression linked to cholesterol transport and efflux	0.017
	Neutrophil degranulation	0.018
	RHOF GTPase cycle	0.019
**ARHGAP5 [53]**	RND3 GTPase cycle	0.019
	RND1 GTPase cycle	0.019
	RND2 GTPase cycle	0.02
**ARL4C [54, 55]**	NR1H2 and NR1H3-mediated signaling	0.021
	Netrin-1 signaling	0.023
	RHOD GTPase cycle	0.025
**UNC5B [56]**	RHOJ GTPase cycle	0.025
	RHOQ GTPase cycle	0.027
	Assembly of collagen fibrils and other multimeric structures	0.028
**TGFB1I1 [57]**	Collagen degradation	0.029
	RHOB GTPase cycle	0.032
	RHOC GTPase cycle	0.034
**HOPX [58]**	RHOG GTPase cycle	0.034
	Collagen formation	0.041
	RAC3 GTPase cycle	0.043
**CDPsig**		
**DUS3L**	RNA Polymerase I Transcription Termination	0.009
	RNA Polymerase I Transcription Initiation	0.013
**AGMAT [59]**	tRNA processing in the nucleus	0.016
	Metabolism of polyamines	0.016
**POP1**	B-WICH complex positively regulates rRNA expression	0.025
	RNA Polymerase I Promoter Escape	0.025
**POLR1A**	Positive epigenetic regulation of rRNA expression	0.029
	NoRC negatively regulates rRNA expression	0.029
**DDX31**	tRNA processing	0.029
	Negative epigenetic regulation of rRNA expression	0.03
**ACTR3B**	RNA Polymerase I Promoter Clearance	0.03

	RNA Polymerase I Transcription	0.031
**NCOA5 [60]**	Epigenetic regulation of gene expression	0.041
