# Supplementary figures and images for: Identification of Prognostic Gene Signatures by Developing a scRNA-Seq-Based Integration Approach to Predict Recurrence and Chemotherapy Benefit in Stage II–III Colorectal Cancer
Source: Int J Mol Sci. 2022 Oct 18;23(20):12460. doi: 10.3390/ijms232012460 (PMC9604003; doi:10.3390/ijms232012460)

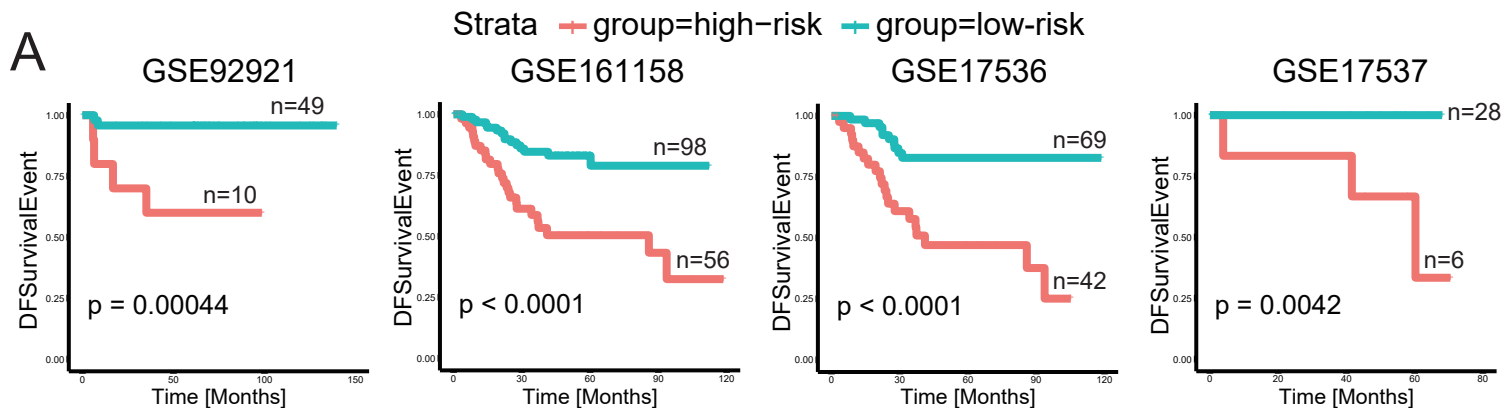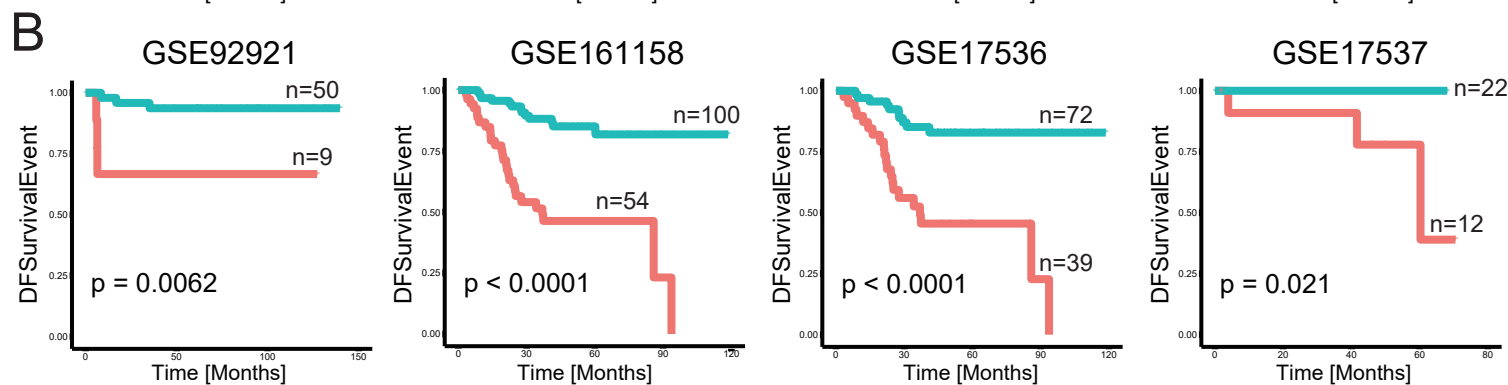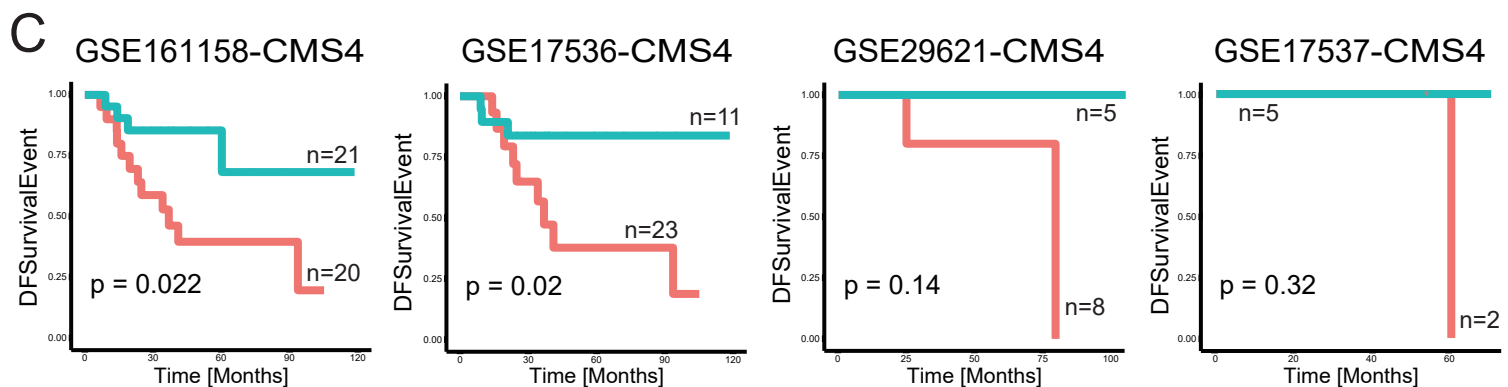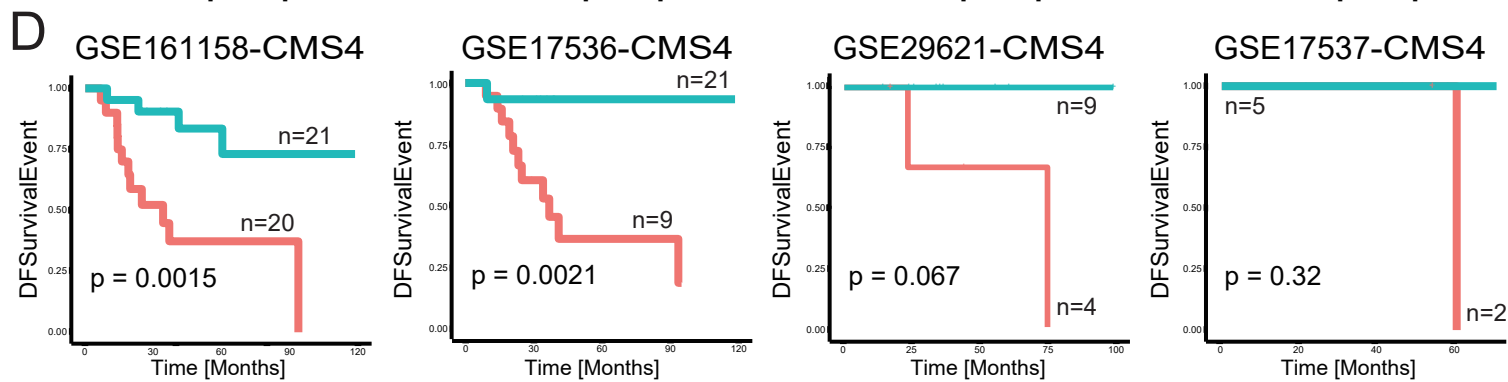

Supplement: Supplementary file 1 [file ijms-23-12460-s001.zip › Supplementary Figure S1.pdf]

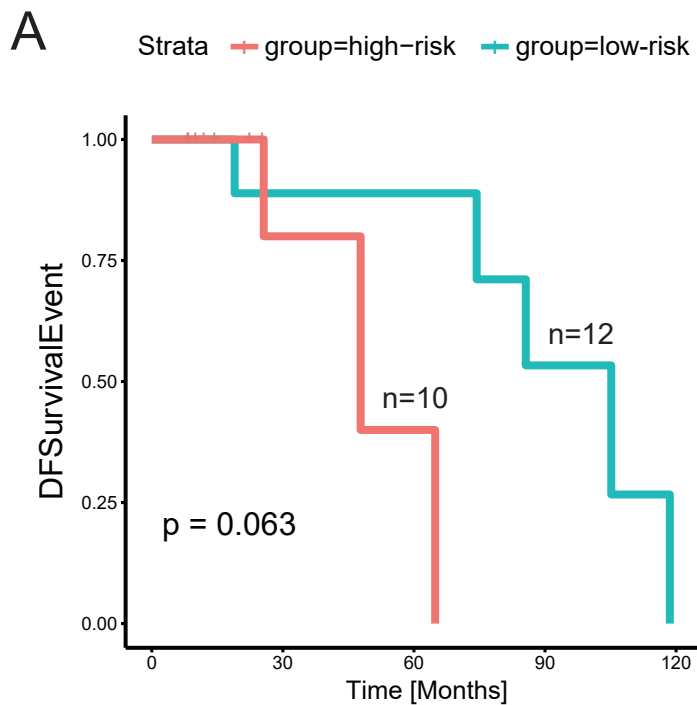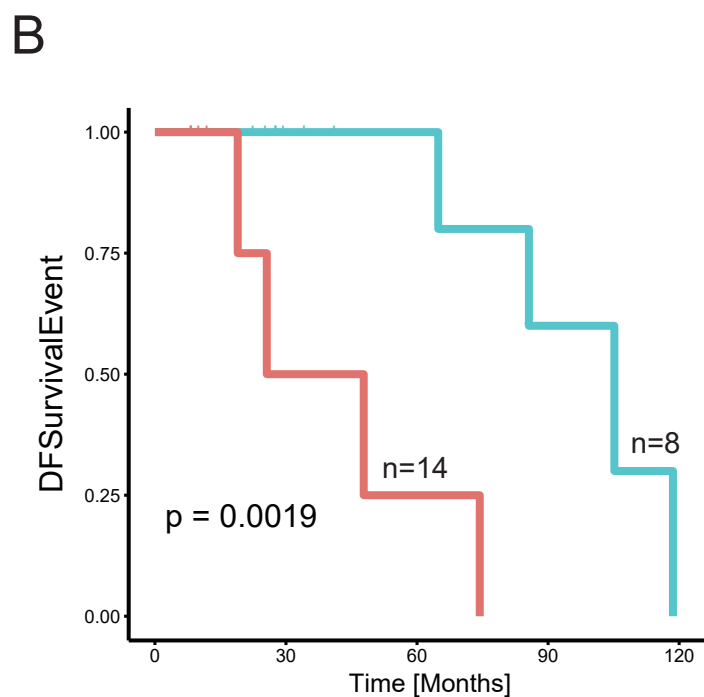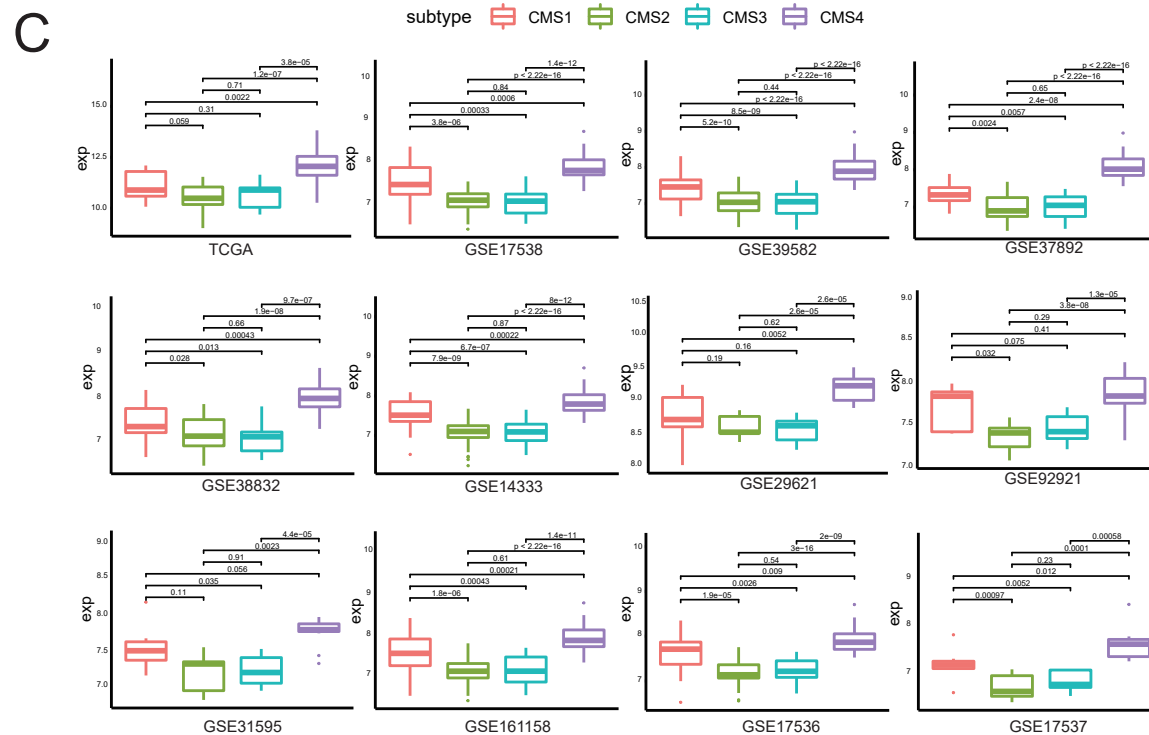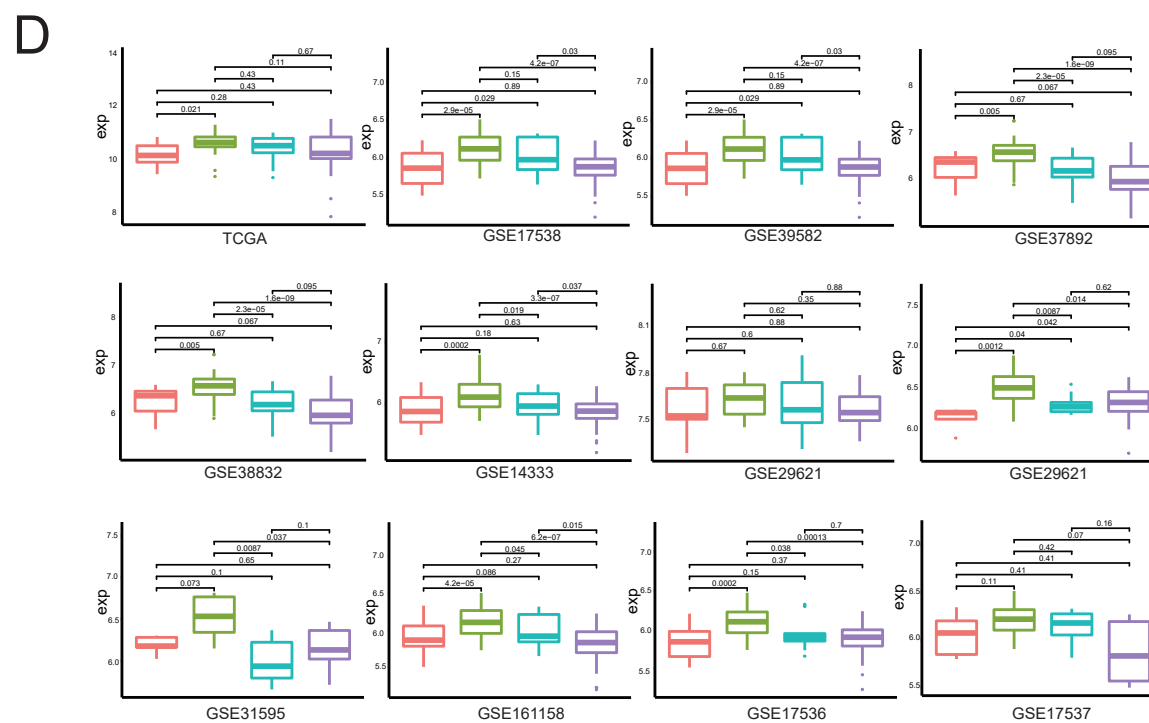

Supplement: Supplementary file 1 [file ijms-23-12460-s001.zip › Supplementary Figure S2.pdf]
